# Population genetics of the Manila clam (*Ruditapes philippinarum*) introduced in North America and Europe

**DOI:** 10.1038/srep39745

**Published:** 2017-01-03

**Authors:** David Cordero, Marina Delgado, Baozhong Liu, Jennifer Ruesink, Carlos Saavedra

**Affiliations:** 1 Instituto de Acuicultura Torre de la Sal, Consejo Superior de Investigaciones Científicas, 12595 Ribera de Cabanes (Castellón), Spain; 2 Instituto de Recerca i Tecnologies Agroalimentaries, Centre de Sant Carles de la Ràpita, Crta. Poble Nou, Km 5.5, 43540 Sant Carles de la Ràpita (Tarragona), Spain; 3 Laboratory of Experimental Marine Biology, Institute of Oceanology, Chinese Academy of Sciences, 7 Nanhai Rd., Qingdao, 266071, China; 4 Department of Biology, University of Washington, BOX 351800, Seattle, Washington 98195-1800, USA.

## Abstract

Globally, the Manila clam (*Ruditapes philippinarum*) stands as the second most important bivalve species in fisheries and aquaculture. Native to the Pacific coast of Asia, it is now well-established in North America and Europe, where its on-going management reflects local economic interests. The historic record of transfers spans the 20^th^ century and suggests sequential movement from Japan to North America, as a hitch-hiker on oysters, and then intentional introduction in Europe, but global genetic data are missing. We have studied mitochondrial DNA and microsatellite markers in nine populations from Asia, North America and Europe. The results from the two types of markers indicated a good concordance of present-day genetic structure with the reported history of clam transfers across continents, and no evidence of relevant concealed introductions from continental Asia in Europe and North America. However, European populations showed a loss of genetic variability and significant genetic differentiation as compared to their American counterparts. Our study shows that in spite of the increasing ease for species to spread out of their native range, in the case of the Manila clam this has not resulted in new invasion waves in the two studied continents.

One of the consequences of global change on marine ecosystems is the sudden introduction of species in new areas, which often show an invasive behavior and interact strongly with native species leading to changes in the ecosystem function^1^,^2^. In some cases, a species is introduced intentionally to expand the economy of the region^3^,^4^, or it creates an economic opportunity in the newly-colonized area^5^. In all situations, the management of the species has to deal with complex interactions between ecosystem health and human economic and social interests. Genetic characterization of species in this context is of special importance to achieve adequate management strategies^6^. Genetic resources of the introduced species can be constrained by the geographic source of the colonizers, founder effects, the size of the founder population (propagule size), the number of independent introductions and the initial demography^7^,^8^,^9^,^10^,^11^. However, species-specific studies focused on genetic structure often fail to align with predictions based on the known introduction history (e.g.: refs 12 and 13). In general, it seems necessary to test specifically in each introduced species the reported introduction history and the genetic structure of the populations in both the native range and in the invaded area in order to obtain a useful population genetic framework that can be applied to management plans.

The Manila clam (*Ruditapes philippinarum*) (Adams and Reeve, 1850), which is one of the most appreciated shellfish species, is a good example of this type of situation. Manila clam culture represented 25% of global mollusk production in 2014. The natural distribution of the Manila clam extends over the western coasts of the Pacific Ocean ranging from the Philippines to Russia^14^. The majority of the world clam production comes from this area, with China as the first worldwide producer (98.9%). However, the species has been introduced in North America and Europe, which now produce 4701 t and 31651 t, respectively (all data from FAO, year 2014; http://www.fao.org/fishery/statistics/global-aquaculture-production/query).

In North America, the Manila clam was recorded for the first time in British Columbia (Canada) in 1936, and it is widely accepted that it came as an unnoticed hitchhiker with oysters imported from Japan^15^. In later years it expanded to the south, reaching its southern limit in California in 1964^16^. In Europe, clams were introduced in France to cope with production problems with the native clam species in 1972–1974, and later they were further introduced in the UK for experimental trials, in both cases from a Canadian stock^3^,^17^. Subsequently, clams of French and British origin were transported to Italy, Spain and Norway^17^,^18^,^19^,^20^. The expansion of the species does not seem to have stopped, as shown by the recent establishment of self-recruiting populations in southern UK and the records from areas located far away from exploitation grounds such as Turkey^21^,^22^.

While the expansion of the Manila clam has been facilitated by its biological traits, such as high fecundity, a long larval phase (ca. 3 weeks), and broad salinity (15–50 PSU) and temperature tolerance (6–30 °C)^23^, the expansion of the species has been boosted by the development of hatcheries for artificial reproduction^24^,^25^. Spat produced in hatcheries have been used frequently to replenish exhausted wild beds or to grow-out in licensed suitable coastal sand plots wherever the species has been introduced.

In North America and Europe the Manila clam became naturalized immediately after its introduction and has shown a typical invasive character. The syndrome of ecological problems caused by invasive species is emerging in places where the Manila clam has established. This include an increase of clam numbers and the displacement of native species^16^,^23^,^26^,^27^,^28^,^29^, but also, in Europe, hybridization with the native clam *R. decussatus* and subsequent introgression^30^,^31^.

In contrast with the expansion experienced in North America and Europe, the situation of the Manila clam in its native range in Asia is characterized by stock reductions attributed to overexploitation and coastal pollution^32^. Solutions taken by Asian fishermen to face clam declines include imports of clams from other regions within Asia, with the consequent population admixture between stocks of China, Korea and Japan at localities where clam fisheries are important^33^,^34^,^35^,^36^,^37^,^38^.

The economic importance reached by the Manila clam, and the problems related to its decline in Asia and its expansion and invasive behaviour in North America and Europe, have boosted scientific studies and integrated management plans^39^,^40^,^41^. But although some genetic studies of populations of Manila clam have been carried out in the last decade at local or regional scales with the aim of helping population management^20^,^34^,^35^,^37^,^38^,^42^,^43^, a global population genetic study is still missing. Moreover, the history of the introduction of Manila clam in America and Europe that is recorded in published reports has never been tested. This is important in these days because global trade facilitates the transport of animals among continents, so there is a real possibility that unrecorded transfers have taken place in North America and Europe. Moreover, the different introduction histories of American and European Manila clam populations might have influenced their current levels of genetic variability. Specifically, even though the number of clams transported with a given oyster import in North America could have been low, the continuous importation of oysters in high quantities over many decades^16^,^44^ probably gave rise to a population that grew on the basis of several successive invasions, and therefore could attain levels of genetic variability similar to the source populations. However, the expansion of Manila clam in Europe has been mediated since the beginning by hatchery spat supply. Since the effective population size of hatchery populations is usually much lower than that of wild populations^45^,^46^, a reduction in genetic variability in the initial population established in Europe would be expected. Furthermore, the continued input of hatchery spat in the exploited populations could have precluded the recovery of the high genetic variability levels^20^,^32^,^33^,^34^,^35^,^36^,^37^,^38^,^42^ typical of wild populations of this species.

Previous studies using genetic markers in native populations indicate that mitochondrial sequences and microsatellites can be used to test the introduction history of the Manila clam and to study the genetic population structure. DNA sequences of the mitochondrial gene *COI* have been studied in populations from China and Japan^33^,^36^. Three clades were found, one of which was characteristic of the Japanese populations, and the other two of the Chinese populations. This geographic distribution revealed a phylogeographic break at the Yellow Sea/Pacific Ocean divide, a common observation in many marine species living in that region that is generally accounted for by historical population subdivision and gene flow restriction caused by the decrease of the sea level during the Pleistocene glaciations^47^. The three clades were mixed in some specific Chinese and Japanese localities, which has been explained by transfer of stocks among Japan, China and Korea for aquaculture^36^. Clear differences between Chinese and Japanese samples were also found using four microsatellites^32^.

In this paper we report a study of the genetic variability of Manila clam populations across continents by using mitochondrial *COI* sequences and microsatellites. Our goals were: 1) to test the reported Japanese origin of American and European populations; 2) to test whether introductions from continental Asia in North America and Europe have taken place; and 3) to test the predictions about the genetic variability of North American and European populations based on their differing colonization histories.

## Results

### Mitochondrial DNA

**Figure 1:**
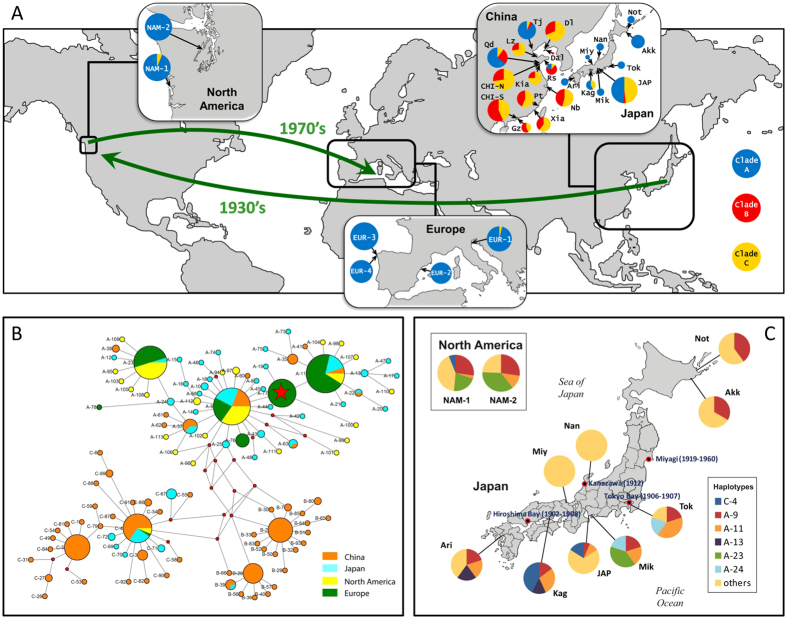
Variability at the mitochondrial *COI* locus in Manila clam populations. (**A**) Frequencies of the *COI* clades A, B and C in populations, based on the data obtained previously^33^,^36^ and in this study. The names of the populations sampled for this study are shown in all-capitals. The sizes of the diagrams are proportional to the number of individuals analyzed. Green arrows indicate the dates and directions of the introduction of the species in North America and Europe. Sampling localities were as follows: CHI-N, Qingdao (China), 36°06′N, 120°13′E; CHI-S, Shenzhen (China), 22°30′N, 114°00′E; EUR-1, Po Delta (Italy), 44°51′N, 12°21′E; EUR-2, Ebro Delta (Spain), 40°46′N, 0°45′E; EUR-3, Ria de Arousa (Spain), 42°38′N, 8°45′W; EUR-4, Ria de Vigo (Spain), 42°19′N, 8°37′W; JAP, Aichi Prefecture (Japan), 34°38′N, 136°53′E; NAM-1, Willapa Bay (USA), 46° 33′N, 123°59′W; NAM-2, Hood Canal (USA), 47°38′N, 122°51′W. (**B**) Phylogenetic network obtained by the median-joining algorithm^67^ showing the evolutionary relationships between the *COI* sequences found in this study and in previous studies^33^,^36^. The size of each circle is proportional to the total haplotype frequency. The colors of the sectors represent the frequency of the haplotype in each of the four regions studied. Red dots represent hypothetical sequences that have disappeared or have not been sampled. A red star marks a haplotype that appeared in relatively high frequency in Europe only. (**C**) Pie charts showing the distribution of *COI* haplotypes in American (boxed) and Japanese populations. Private alleles were pooled in each population. The localities that were the sources of oyster imports in N. America in the 20th century are shown in red dots, and the time periods in which the documented importations took place are given in parentheses. ***Maps have been obtained from Google Maps. Map data ©2016 Google, SK Telecom, Zenrin***. Pie charts were drawn with Microsoft Excel. Maps were modified with Adobe Illustrator 14.0.0 and Adobe Photoshop Extended 11.0.2.

We sampled 9 populations, two from China, one from Japan, two from North America and four from Europe (Fig. 1A). We obtained a sequence of 581 bp of the mitochondrial COI gene from 253 individuals. To increase the scope of the analysis, we pooled our data with those of previous studies^33^,^36^ resulting in a total of 423 sequences from 28 populations.

#### Haplotype and clade frequencies

We found a total of 113 haplotypes, from which 82 were unique (Supplementary Table S1). Sixty six haplotypes had been already described^33^,^36^, and 47 were new to this study, mainly coming from American and European samples. The new sequences have been deposited in the EMBL database under accessions LT600424-LT600470. The evolutionary relationships among *COI* haplotypes and their frequencies by regions are represented in a phylogenetic network in Fig. 1B. Three clades (A, B, C) were apparent, as previously found^36^. Our two Chinese samples showed clades B and C only, and our Japanese sample showed three clades, with clade A being the most abundant. The overwhelming majority of the haplotypes found in America and Europe were of clade A, with just a few individuals carrying clade C haplotypes in these regions. A crucial detail was that no haplotype was found in any American or European population that, being present in China, was not present in Japan. We noticed an abundant (27%) haplotype (A-77) in Europe that was absent from American and Asian populations (Fig. 1B). American populations showed haplotype A-23 in intermediate frequency (29%). This haplotype appeared also in a Japanese population (Mik) (Fig. 1C).

#### Haplotype and nucleotide diversity

**Figure 2:**
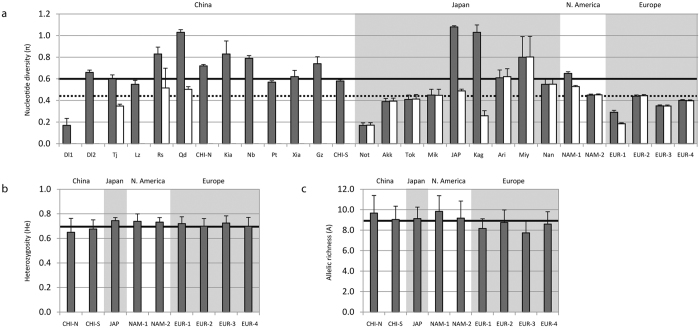
Genetic variability in Manila clam populations. (**a**) Nucleotide diversity estimated from the whole set of *COI* sequences (grey bars), and from the clade A sequences only (white bars). Lack of a white bar in some populations indicates the absence of clade A, not zero diversity. The averages across populations are shown as a horizontal thick line (all clades) or a dashed one (clade A). (**b**) Heterozygosity at microsatellites. The horizontal black line shows the average across populations. (**c**) Allelic richness at microsatellites. In all figures, alternate white and gray backgrounds separate groups of populations belonging to different geographic regions (China, Japan, North America, Europe).

Our analyses confirmed the high levels of haplotype diversity (*H*_*d*_) and low levels of nucleotide diversity (*π*) found previously^36^ in Asian populations, and extended them to America and Europe. *H*_*d*_ ranged from 1.00 (in four Asian populations) to 0.69 (EUR-1) (Supplementary Table S2). By regions, Asia showed the highest values of *H*_*d*_ (0.80–1.00) and Europe the lowest (0.69–0.82), with American populations showing intermediate values (0.79 and 0.89). A similar trend was found in the case of *π* (0.17–1.08 in Asia, 0.45–0.65 in America, and 0.29–0.44 in Europe; Fig. 2a). Among clades, *H*_*d*_ and *π* were slightly higher for clade A (Supplementary Table S2). The highest *H*_*d*_ and *π* values for this clade were found in Japan and the lowest in Europe, with a few exceptionally low values found in some of the Japanese populations (*e.g*.: Not, Kag), taken from a previous study^33^, which had very low sample sizes (N = 5–7) (Supplementary Table S2 and Fig. 2a).

#### Neutrality tests

Neutrality tests (Supplementary Table S2) rendered significant (P < 0.05) values for Tajima’s *D* in only 1 population, indicating no significant departures from neutrality in the *COI* data set.

#### Population genetic differentiation

Significant (P < 0.05) pair wise *F*_*ST*_ ranged between 0.041 and 0.246, and in most cases were significant after a sequential Bonferroni test (Table 1). No significant differentiation was found between pairs of European populations and between the two American populations. Chinese and European populations were the most differentiated, as indicated by pairwise *F*_*ST*_ values ranging from 0.111 to 0.246. Population pairs involving China and America gave slightly higher *F*_*ST*_ values than those involving China and Japan, and the Japanese JAP population was more differentiated from European than from American populations. These results indicate a gradient of genetic differentiation from China to Europe, as expected from the reported clam introduction history. The European EUR-2 sample was not significantly differentiated from both American samples.

We obtained a total *F*_*ST*_ value of 0.109 (Table 2). Within each region the highest variation among populations was found in China (*F*_*ST*_ = 0.063, P < 0.001) and Japan (*F*_*ST*_ = 0.041, P < 0.01), followed by Europe (*F*_*ST*_ = 0.034, P < 0.05).

AMOVA^48^ and hierarchical F-statistics gave very similar results, and here we present F-statistics only, because they allow a direct comparison with microsatellites. Using a hierarchical model with four regions (Table 2), we obtained a total *F*_*ST*_ of 0.126, and interregional differentiation accounted for the majority of interpopulation variability (*F*_*CT*_ = 0.081). A hierarchical model was also used to extract the among-population, within-regions component in genetic differentiation tests between pairs of regions. China and Europe were the most differentiated regions (*F*_*CT*_ = 0.132; P < 0.001). No significant differentiation between Japan and America was found (*F*_*CT*_ = 0.033, P > 0.05). America and Europe showed a high but non-significant interregional differentiation (*F*_*CT*_ = 0.093).

### Microsatellites

The 7 polymorphic microsatellites yielded a total of 114 allelic variants in the 9 scored populations (Supplementary Table S3). Null alleles were detected in all populations at loci K22 and A54, with frequencies ranging from 0.16 to 0.42, and at locus K8 in two populations (CHI-S and NAM-1) with estimated frequencies of 0.06 and 0.08 respectively (Supplementary Table S3). None of the Linkage disequilibrium tests performed on the 189 gene pair comparisons were significant after sequential Bonferroni correction. Significant departures from Hardy Weinberg equilibrium were found in 15 tests after Bonferroni correction, with *F*_*IS*_ values ranging from −0.132 in EUR-4 to 0.183 in NAM-1 (Supplementary Table S4), but no clear pattern of variation across populations or loci was observed.

#### Genetic diversity

Microsatellite allele number (*N*_*a*_) and expected heterozygosity (*H*_*e*_) showed average values of 9.5 ± 0.3 and 0.7 ± 0.01, with little variation among populations, except slightly lower diversities in China (Supplementary Table S4 online and Fig. 2b). Allelic richness (*A*) over loci ranged from 9.8 ± 4.1 in NAM-1 to 7.7 ± 3.2 in EUR-3, with the lowest values observed in European populations (Fig. 2C and Supplementary Table S4).

Private allelic richness (*A*_*p*_) was lowest in European populations and CHI-S (Table 3). The lowest *A*_*p*_ was found at EUR-3. Estimates for each region showed that *A*_*p*_ was highest in America and lowest in Europe (Table 3).

#### Population genetic differentiation

**Figure 3:**
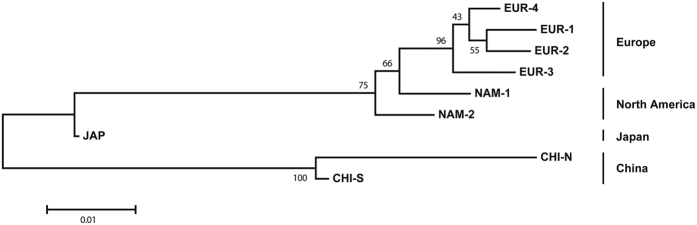
Neighbour joining tree showing the relationships among the 9 populations of Manila clam based on Nei’s *D*_*A*_ distance estimated from allele frequencies at 7 microsatellite loci. Bootstrap proportions obtained from 10,000 replications are shown above branches.

The neighbor-joining tree based on microsatellite *D*_*A*_ distances^49^ is shown in Fig. 3. Populations from China formed a separate cluster, with the highest distance and bootstrap support. European populations formed another cluster with high bootstrap support. American populations were basal to the European cluster. The Japanese population appeared equidistant between the Chinese and the American.

Significant *F*_*ST*_ values were obtained in 24 out of 36 pair-wise tests, of which 22 were significant after Bonferroni correction (Table 1). Non-significant values fell on pairs of American and European populations and on comparisons inside these regions. The total *F*_*ST*_ was 0.045 (P < 0.001) (Table 2). In agreement with pair-wise *F*_*ST*_ values, when *F*_*ST*_ was computed within each region it was non-significant in America and Europe, and it was low but significant in China.

The hierarchical analysis of variance based on a model of four regions (Table 2) showed that the variability among regions (*F*_*CT*_) accounted for 5.4% of the total variation among populations. *F*_*CT*_ values obtained with hierarchical analysis of F-statistics for two-region models indicated that the most differentiated regions were both Europe and America with respect to China, and the less differentiated were America and Europe, as expected from the reported history of clam introductions.

**Figure 4:**
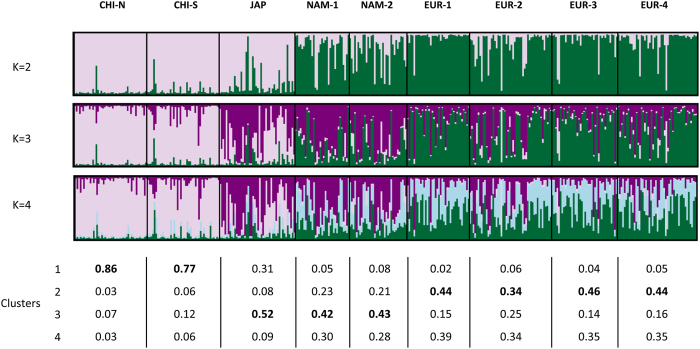
Bayesian model-based cluster analysis of individual genotypes at 7 microsatellite markers in 9 populations of Manila clam. Estimated membership fractions for each individual and population are shown for K = 2, K = 3 and K = 4 clusters. Membership fractions corresponding to each cluster in each population for K = 4 are given below the chart, with the most common cluster marked in bold. For K = 4, clusters 1, 2, 3 and 4 are shown in pink, green, purple and blue, respectively.

Results of Bayesian model-based clustering (BMBC) of genotype data^50^ are shown in Fig. 4. The Evanno *et al*. method^51^ indicated a most probable K of two clusters (Supplementary Fig. S1a), but the results of the Pritchard method showed a posterior probability of data reaching a plateau in K values of 2, 3 and 4, suggesting a more complex hierarchical structure (Supplementary Fig. S1b). We present therefore the results of the analysis for K = 2–4 (Fig. 4). With K = 2, individuals were classified geographically in two groups. One group was formed by the individuals from Asian populations and characterized by a high frequency of cluster 1. The other group was formed by the individuals from the other regions and was characterized by a high frequency of cluster 2. With K = 4, regions showed a characteristic pattern of cluster frequencies (Fig. 4). For example, cluster 1 was more abundant in China and almost absent in the other regions. Cluster 3 was most abundant in Japan and America, and almost absent from China, and clusters 2 and 4 were most abundant in Europe.

#### Assignment tests

Results of assignment tests are given in Table 4. In Asian and American populations most individuals (63% to 82%) were assigned to the populations where they were sampled or to a population in the same region (3% to 23%), although a small fraction (less than 10%) was assigned to populations in a neighbor region, and 7 individuals were assigned to populations in a non-neighbor region. A few individuals sampled in America were assigned to European populations. In Europe the majority of the individuals were assigned to populations where they were not sampled, although usually within the same region. However, a substantial proportion (30–44%) of individuals sampled in European populations were assigned to American populations, with the highest proportion observed in the EUR-2 population. Moreover, 2 European individuals were assigned to JAP and 2 to CHI-N. Assignment of individuals to regions showed a similar pattern (Table 4). Values o the *D*_*LR*_ statistic^52^ higher than 3 were observed in 8 cases, all comprising comparisons between Chinese populations and American or European populations. This result indicates that the power to distinguish an individual from a given reference population in the assayed sample was sufficiently high (>50%) as to allow relatively robust inferences from these comparisons.

#### Non-random association of mitochondrial haplotypes and microsatellites

This test could be carried out only in the JAP population, where mitochondrial clades A and C were in sufficient frequencies. We tested the difference in the estimated membership fraction of the genotype cluster 3, obtained from the BMBC analysis, in clams carrying clade A *COI* haplotypes and in clams carrying *COI* haplotypes of other clades (Supplementary Table S5). A Student *t* test indicated that differences between the two groups were not statistically significant (*P* = 0.65).

## Discussion

Population genetics of Manila clams in their native range has been described elsewhere, but a specific discussion of the results obtained from our Asian samples is necessary before using them as a reference in the study of American and European populations. Previous studies^33^,^36^ reported the presence of mitochondrial clades B and C in some Japanese populations, and of clade A in some Chinese populations, and they attributed this observation to the effect of clam transplantation between the two countries for aquaculture. This explanation was supported by the concentration of the anomalous clade presence in the areas of most intensive clam exploitation. We have found only clades B and C in our Chinese samples, which suggests that they are composed of Chinese genetic backgrounds exclusively. However, we found a mixture of clades A, B and C in our Japanese sample (JAP), which comes from an area (Ise Bay) of traditional clam fishing. Therefore it is highly probable that the presence of B and C clades in JAP is due to aquaculture practices. This conclusion is supported by the comparison of our data with those of previous studies. Our Japanese sample comes from the same area as the Mik population analysed previously^33^. However, the Mik sample had clade A haplotypes only (Fig. 1c). This suggests that the population from which the JAP sample was taken might have been originated by a transplantation to Ise Bay of clams collected elsewhere, which had a high proportion of genes of Chinese ancestry. The presence of haplotypes C-4 and A-11 in JAP suggests that the collection place of the putative transplanted clams could be located in SE Japan, since those haplotypes are characteristic of this area (Supplementary Table S1).

Microsatellite data also support this view. We have found that most microsatellite alleles were present in the samples from both regions, and only a small proportion were exclusive of Japanese or Chinese samples, in agreement with previous studies^32^. However, the BMBC analysis of genetic structure with K = 4 showed that there is a clear differentiation at nuclear loci between China and Japan which affects microsatellite clusters 1 and 3. The high frequency of cluster 1 observed in the clams from JAP indicates that the Chinese ancestry is present in the nuclear genome of these clams, in agreement with the *COI* results. The absence of significant non-random association between nuclear alleles and mitochondrial haplotypes shows that the admixture between Chinese and Japanese genetic backgrounds in JAP occurred several generations ago, otherwise there would not have been time enough for the associations established when founding the population to disappear^53^.

The *COI* data support the Japanese ancestry of North American populations. All the individual clams from North America analysed in this study, with only two exceptions, carried clade A haplotypes, as expected if they originated from Japan. In the case of the two individuals that carried haplotypes of other clades, the haplotype was C-4 in both cases. This was the most abundant haplotype of clade C, and has been documented in some localities from Japan in previous^33^,^36^ and in this study (Supplementary Table S1). Therefore, the presence of this haplotype in our North American samples can be explained by assuming that they were present in the clam samples transported from Japan to North America with oyster imports which originated the American populations in the 20th century^15^,^16^.

The Bayesian model-based clustering (BMBC) analysis based on microsatellites also provided support to the Japanese origin of North American Manila clams. In this case the interesting observation is that cluster 3, which was the most common in the Japanese sample, but was rare in China, was also the most common in the American populations. Similarly, cluster 1, which was the most common in China, was almost absent from North America (Fig. 4). The clustering of North American and Japanese populations based on genetic distances provide additional support to the reported introduction history (Fig. 3).

The populations of Manila clam first recorded in America are supposed to have originated from nonintentional introductions, as a carry over with the Pacific oyster imports from Japan in the 1920’s to 1970’s^44^,^54^. There is a relatively good record of oyster importations into the USA, which originated in several localities (Fig. 1c). Miyagi, in the NE of Japan (some 600 km away from Mik) is the most probable source of the majority of the oyster imports in the times in which the Manila clam invaded N. America^44^, although the absence of a sample from this locality in this and in previous studies impedes testing this origin. However, some considerations can be done. The American populations share many haplotypes with the populations of the Pacific coast of Japan, which supports their origin in this area (Fig. 1c). On the other hand, the populations from the Sea of Japan carry completely different haplotype sets (Fig. 1c), which rejects west Japanese localities as a source of the American Manila clams. The two American samples are characterized by the presence of haplotype A-23. Out of N. America, this haplotype was found only in the Mik population in Japan (Fig. 1c). This suggest that the central Pacific coast of Japan could be the origin of at least part of the American founder population. However, it is also possible that this haplotype appeared by mutation in North America after the colonization, and its presence in Japan is due to a recent, unrecorded introduction of clams from North America for restocking the Mik region.

Our results also provide support for the North American origin of the European populations. In the case of *COI*, the presence of haplotypes of clade A, which was typical of American populations, was almost exclusive in the European samples. The only exception, an individual from EUR-1, carried a clade C haplotype (C-4), which was also present in very low frequencies in America (Fig. 1b). However, all the haplotypes that appeared in Europe and in America, have also appeared in Japan, so the *COI* data are also compatible with a direct introduction from Japan into Europe. The microsatellite data were more conclusive. The pairwise *F*_*ST*_ values between Europe and American populations were several times lower than those found between the European samples and the Japanese one (Table 1). In concordance, European populations clustered with the American samples in the distance tree (Fig. 3). Moreover, the BMBC analysis showed that American and European populations shared the presence of genotype clusters 2 and 4 in intermediate frequencies, while these clusters were very rare in Japan and China (Fig. 4). Finally, the great majority of the European clams which were not ascribed to their own region in the assignment tests were assigned to an American population (Table 4).

While our data indicate that the majority of the gene pool of Manila clam populations from North America and Europe have an eastern Japanese ancestry, industrial development of Manila clam aquaculture in North America and Europe in the last 30 years could have led to concealed clam introductions from geographic sources other than Japan. These introductions should have left a genetic footprint. A potential source is China, where clam aquaculture is strong. However, our results indicate that the great majority of European individuals are of European, American or Japanese ancestry only. In the few cases of European clams that carried *COI* haplotypes that were present in China, those haplotypes were also found in Japan and in America, thus providing a reasonable, alternative explanation for their presence in Europe based on their presence in the American founders of the European population. However, the assignment tests based on microsatellite data discovered a few individuals in European populations showing a possible Chinese nuclear genome ancestry. This is not a direct evidence for an introduction from China, since these cases could be explained as resulting from the normal shuffling of genotypes in recombinant genomes of mixed Chinese and Japanese backgrounds of the founders. Moreover, the high homoplasy typical of microsatellites^55^ can result in alleles of equal length appearing in distant localities. In any case, a more intensive sampling of populations and loci would be necessary to completely discard this possibility.

Concealed introductions of clams from Japan and North America in Europe after the initial introduction in the 1970’s would be difficult to distinguish due to the low power of genetic differentiation and assignment tests in contexts of low genetic differentiation. However, they have occurred. While preparing this paper a hatchery dealer informed us that adult clams from North America had been introduced in at least two Spanish hatcheries in the past 15 years to be used as broodstock. It is possible that the EUR-2 population is reflecting this recent introduction. The genetic profile of this population has a high similarity to North American populations. For example, in the BMBC analysis of genetic structure, the frequency of cluster 3 in EUR-2 was clearly higher than in the remaining European samples (Fig. 4). Moreover, nearly as many individuals from this population were assigned to North America as to Europe when the assignment test was applied (Table 4).

Values of microsatellite allelic richness observed in the four European populations were lower than those of the American and Asian populations. In the case of heterozygosity (*H*_*e*_) the lowest values appeared in the two Chinese samples, but the European populations showed lower values than the two American and the Japanese samples (Fig. 2b). Moreover, the European samples showed a much lower frequency of private alleles than the North American and Japanese samples. These results indicate a reduction of variability at the nuclear genome in European clam populations as compared to their native range and in North America (Fig. 2b and c). Mitochondrial DNA variability showed a similar trend, but in this case it was less clear due to low sample sizes in some Chinese and Japanese populations (Dl1, Not) (Supplementary Table S2), and to the admixture of Chinese and Japanese clams in some culture areas (JAP, Kag). However, if we exclude those populations, we obtain the same picture that was obtained from microsatellites. The four European populations showed lower indices of total nucleotide diversity than American populations at the mitochondrial locus (in total and at clade A only), and also than most Japanese samples (Fig. 2a).

The picture emerging from these results is one of lower genetic variability in European populations. Given the history of clam transfers across continents, the most probable cause is a founder effect. Founder effects associated to the invasion of new areas have been described either in natural expansions or in human -mediated expansions (invasions)^11^. Although genetic variability can be recovered after the initial bottleneck in recently founded populations if the population expands^56^,^57^, in the case of Manila clams in Europe, the expansion has been mediated to a great extent by the release of spat obtained in commercial hatcheries. Hatchery spat usually have less variability than wild spat (e.g.: ref. 45). Therefore, it is probable that the continuous input of low-variability clams from hatcheries is responsible for the low genetic variability that we are observing today, almost 50 years after the first introduction of the species in Europe and after an expansion along wide areas of the European coast.

Our microsatellite results have shown that populations of Manila clam are characterized by small overall differentiation, a higher interregional differentiation (*F*_*CT*_) between China and Europe than between Europe and North America, and a lack of genetic differentiation within Europe and North America. These results are congruent with the short time elapsed since the introduction of the Manila clam in these two regions, the long larval period that facilitates gene flow within the regions, and the aquaculture practices that have been developed after the introductions in N. America and Europe and favour the genetic mixing of populations. Mitochondrial data agree with this picture in general, although the values of population differentiation are higher for *COI* than for microsatellites as expected from its uniparental mode of inheritance^58^,^59^. However, *COI* data contrast with microsatellites in showing higher differentiation between Europe and N. America than between N. America and Japan, which split earlier. Moreover, one haplotype (A-77) that was in relatively high frequency in European populations was absent from the American and the Japanese populations (Fig. 1b and Supplementary Table S1), although this should be confirmed by scoring a larger number of populations in N. America and Japan. Since mitochondrial DNA has a lower effective population size than microsatellites, it is expected that genetic drift and bottlenecks act more strongly on *COI*. Our observations of higher differentiation between Europe and N. America at *COI* support the occurrence of a strong founder effect in the European population.

In conclusion, we have shown that the reported history of introductions of Manila clam populations in North America and Europe in the 20^th^ century explains the current genetic population structure. The North American and European populations share a genetic pool which shows a Japanese ancestry, and the contribution of Chinese populations to this pool should have been rare or nonexistent. Our observation of a general absence of genetic footprints from continental Asian clams suggests that neither the commercial basis of clam aquaculture nor the biological characteristic of the species have facilitated this expansion along almost a century. Detailed economic and biological studies on why this limitation has occurred would be interesting to help in understanding, preventing and managing uncontrolled expansion of other marine species. More recent introductions from Japan to North America, or from Japan or North America to Europe, cannot be detected statistically with our data, but there are clues that some European populations have experienced them. Finally, we have confirmed the predictions, derived from the available information on the introduction history, that genetic variability has been reduced in European populations as a consequence of a founder effect and of propagation mediated by farmed clams.

## Materials and Methods

### Sampling, DNA extraction, PCR and sequencing

Nine populations of Manila clam *Ruditapes philippinarum* were sampled from Asia (China and Japan), Pacific North America and southern Europe (Fig. 1a). Twenty to forty animals were collected in each locality, and dissected upon arrival in the laboratory. Samples of adductor muscle, gill and mantle tissues were stored in 90% ethanol until the genetic analysis was performed.

DNA extraction was carried out by boiling a piece of tissue in a 10% Chelex 100 preparation of 200–400 mesh (Biorad) during 20 minutes.

### Mitochondrial DNA amplification and sequencing

The universal forward primer LCO1490^60^ and an ad hoc reverse primer, COI-CB (5′-gaatctcctaacctgtwggatcaa-3′), were used. PCR was carried out in 40 μl reactions with 4 μl of 1:10 diluted template DNA, 2 mM of MgCl2, 0.8 μM of each primer, 0.2 mM of each dNTP and 1 U Taq DNA Polymerase (Invitrogen) in the buffer supplied by the manufacturer. PCR conditions were set to an initial denaturation of 2 min at 94 °C, followed by 35 cycles consisting of 30 s at 94 °C, 30 s at 48 °C, and 1 min s at 72 °C, and a final 1 min step at 72 °C. UltraClean PCR Clean-Up Kit (MO BIO) was used for purification of PCR products. Sanger sequencing was carried out at the genomics section of the SCSIE in the University of Valencia (Spain), using a ABI 3730 automated sequencer.

### Microsatellite selection and genotyping

After initial trials, seven microsatellites were selected for the present study on the basis of the absence or very low and manageable occurrence of artifacts such as allele dropout, stuttering or null alleles: Asari16, Asari24, Asari54, Asari62 and Asari64^61^, and KTp8 and KTp22^62^. Microsatellite amplification was carried out in total volume of 20 μl, containing a mixture of 1 μl of template DNA, 10 μM primers (Applied Biosystems), 2.5 mM MgCl2, 1xBuffer (Invitrogen), 10 mM of each dNTP and 0.75 units of *Taq* polymerase. The forward primer was fluorescently labeled with one of NED, VIC, PET or FAM fluorochromes. Each PCR started with a denaturation step of 3 minutes at 94 °C, followed by 35 cycles of 30 seconds each, consisting of denaturation at 94 °C, annealing at 58 °C and extension at 72 °C. A final step of 72 °C was carried out during 2 minutes. The PCR products were electrophoresed on an automated sequencer (ABI 3730 or ABI3730XL) at the genomics facilities of the SCSIE (University of Valencia, Spain).

### Mitochondrial DNA data analysis

The sequences obtained were edited with Bioedit^63^ and aligned using Clustal W^64^. The number of haplotypes (*h*), their frequencies, number of segregating sites (*S*), measures of haplotype diversity (*Hd*) and nucleotide diversity (*π*), and their standard deviations, were determined with the software package DnaSP v5^65^,^66^.

The evolutionary relationships among COI sequences were represented with a phylogenetic network constructed with the program Network (fluxus-engineering.com), using a median-joining algorithm^67^ for multi-state sites. The parameter epsilon was set to 10 to explore all possible shortest trees, subsequently unnecessary median vectors and links were deleted with the MP option^68^.

Pair-wise *F*_*ST*_, hierarchical F-statistics^69^ and AMOVA were used to study the genetic differentiation of populations with the software package Arlequin v.3.5^48^. In hierarchical F-statistics, the total genetic differentiation among populations (*F*_*ST*_) was partitioned in among-regions (*F*_*CT*_) and among-populations, within-regions (*F*_*SC*_) components. The significance of the statistics was tested with a non-parametric permutation approach run for 30000 permutations.

### Microsatellite genotyping and data analysis

Genotyping was performed with the software GeneMapper v.4.1 (Applied Biosystems). Allele calling was achieved by adjusting the automated allele binning to a known internal size standard run alongside the PCR fragments. Low quality genotyping scores were revised manually. The program ALLELOGRAM^70^ was used for normalization of data from different runs. Internal controls were used in each run of samples for the automated binning. Genotyping errors like non-amplified alleles, allelic dropout, short allele dominance and stutter products were detected with the program Micro-checker^71^.

Allelic frequency estimation and Hardy Weinberg (HW) equilibrium tests were performed with the program Genepop'007^72^. The frequencies of null alleles were estimated, and the observed allele and genotype frequencies adjusted, with the program Micro-Checker^71^, using the Brookfield 2 estimator^73^. Exact P-Values were obtained by means of the Markov chain algorithm^74^, with 10000 dememorization steps, 20 batches and 500 iterations per batch. Estimates of *F*_*IS*_ were obtained across loci and population^69^. Expected heterozygosity^65^ and allele number per locus and populations were calculated with the program Arlequin ver 3.5^48^. Allelic richness per locus, population and overall populations, was obtained with the program Fstat v.2.9.3^75^. A linkage disequilibrium test was done for each pair of loci in each population and across all populations by means of a G-test implemented in the program Genepop ‘007^72^. Significant levels were obtained with a Markov chain algorithm^76^ with 10000 dememorization steps, 100 batches and 5000 iterations per batch.

We studied the private allele richness (*A*_*p*_) across populations by means of a rarefaction procedure implemented in the program ADZE^77^. We used the extended rarefaction method^78^, which allows correction for sample size differences, and computed the generalized private allele richness statistic^77^.

Genetic differentiation and population subdivision were studied with pairwise *F*_*ST*_ and hierarchical F-statistics, as described for mitochondrial marker. We also estimated *D*_*A*_ distances^79^, which were used to construct a neighbor-joining tree^80^. When working with a small number of microsatellite loci, this distance measure has higher probabilities of obtaining a more reliable population trees than other distances^81^. Statistical support was attained with a Bootstrap test of 10000 replicates. All calculations were done with the program POPTREE2^82^.

A different analysis of population genetic structure was carried out by means of a Bayesian model-based clustering (BMBC) method with the software STRUCTURE, version 2.3.4^50^. By this method we obtained the estimates of the membership fractions of each individual to the inferred ancestors of the current populations. Individual genotypes of each population were analyzed under a model of admixture with correlated allele frequencies among populations, and no prior information on sample locations was assumed. With this model any of the inferred clusters is allowed to be the ancestor of an individual’s genome fraction, and genotypes of source clam populations are equally involved in the inference of the population structure of recent introductions. Data were analyzed with clustering models from K = 1 to K = 10, with 8 replicates of 100000 iterations and previous burning of 150000. Assignment of the most probable clustering of the inferred ancestral populations was analyzed with two methods^51^,^83^ implemented in the website Structure Harvester^84^. Results of the 8 replicates obtained with STRUCTURE were ordered and averaged with CLUMPP^85^ and graphically represented with DISTRUCT^86^.

Assignment tests were performed on individual multilocus genotypes in order to estimate the probabilities of individual clams to be resident to a reference population. Individual assignation likelihoods were computed with the program GENECLASS2^87^ using the frequency criterion based on Paetkau *et al*. formula^52^. For the alleles that were absent in reference populations but present in the to-be-assigned sample, the allelic frequency in the reference population was set to 0.01. In order to obtain the probabilities of residence, assignation likelihoods were compared with the distribution of likelihoods obtained for the population under the resampling Monte Carlo method^52^. The *D*_*LR*_ statistic, a measure of the power of successful assignment^88^, was also calculated. Values higher than 3 indicate 50% power to distinguish an individual from a given reference population in the assayed sample.

## Additional Information

**Accession codes**: GenBank accessions of COI sequences are LT600424-LT600470.

**How to cite this article**: Cordero, D. *et al*. Population genetics of the Manila clam (*Ruditapes philippinarum*) introduced in North America and Europe. *Sci. Rep.* **7**, 39745; doi: 10.1038/srep39745 (2017).

**Publisher's note:** Springer Nature remains neutral with regard to jurisdictional claims in published maps and institutional affiliations.

## Supplementary Material

Supplementary Tables and Figures

## Figures and Tables

**Table 1:** Pairwise *F*_*ST*_ values based on microsatellite data (above diagonal) and on *COI* haplotype frequencies (below diagonal).

	CHI-N	CHI-S	JAP	NAM-1	NAM-2	EUR-1	EUR-2	EUR-3	EUR-4
CHI-N	0	**0.013**	**0.061**	**0.110**	**0.111**	**0.117**	**0.111**	**0.108**	**0.109**
CHI-S	**0.071**	0	**0.044**	**0.076**	**0.075**	**0.090**	**0.084**	**0.079**	**0.080**
JAP	**0.041**	**0.089**	0	**0.024**	**0.018**	**0.038**	**0.031**	**0.030**	**0.033**
NAM-1	**0.074**	**0.138**	**0.055**	0	−0.002	0.007	0.003	0.005	0.002
NAM-2	**0.126**	0.198	**0.105**	0.005	0	**0.013**	0.003	**0.009**	**0.006**
EUR-1		**0.246**		**0.168**	**0.191**	0	−0.007	−0.001	−0.002
EUR-2	**0.110**	**0.186**	**0.079**	0.021	0.000	**0.096**	0	−0.001	−4.8E-04
EUR-3	**0.168**	**0.240**	**0.129**	**0.141**	**0.126**	0.047	0.023	0	−0.003
EUR-4	**0.143**	**0.217**	**0.118**	**0.092**	**0.0720**	0.031	0.004	0.005	0

Statistically significant (P < 0.05) values appear in bold, and those significant after applying Bonferroni correction are underlined.

**Table 2:** Results of F-statistics and hierarchical F-statistics analysis for models of two and four regions for *COI* and microsatellites.

	*COI* (28 populations)			Microsatellites (9 populations)		
	*F*_*SC*_	*F*_*ST*_	*F*_*CT*_	*F*_*SC*_	*F*_*ST*_	*F*_*CT*_
Total		0.109***			0.045***	
China		0.063***			0.015***	
Japan		0.041**			—	
America		0.005			0.001	
Europe		0.034*			0.001	
China-Japan	0.057***	0.081***	0.026*	−0.003	0.028**	0.031**
China-America	0.054***	0.127***	0.077**	0.008**	0.091***	0.084***
China-Europe	0.056***	0.181***	0.132***	0.004*	0.093***	0.089***
Japan-America	0.033*	0.065***	0.033	0.001	0.028***	0.028
Japan-Europe	0.043**	0.120***	0.081**	0.001	0.040***	0.040***
America-Europe	0.024*	0.115***	0.093	0.001	0.009**	0.008***
China-Japan-America-Europe	0.049***	0.126***	0.081***	0.004*	0.057***	0.054***

^*^P < 0.05; ^**^P < 0.01; ^***^P < 0.001.

**Table 3:** Estimates of the mean private alleles richness per locus (*A*_*p*_), computed by the extended rarefaction method^78^.

	*A*_*p*_
Populations	
CHI-N	0.567 ± 0.32
CHI-S	0.041 ± 0.03
JAP	0.565 ± 0.21
NAM-1	0.750 ± 0.27
NAM-2	0.388 ± 0.23
EUR-1	0.023 ± 0.02
EUR-2	0.098 ± 0.05
EUR-3	0.010 ± 0.01
EUR-4	0.234 ± 0.12
Regions	
China	1.21 ± 0.26
Japan	1.14 ± 0.32
America	1.43 ± 0.50
Europe	0.60 ± 0.22

Standardized sample sizes of 62 and 72 individuals were used, respectively, for populations and regions.

**Table 4:** Results of assignment tests using the algorithm of Paetkau *et al*. 52.

Population	Proportion of individuals assigned to populations									Proportion assigned to regions			
	CHI-N	CHI-S	JAP	NAM-1	NAM-2	EUR-1	EUR-2	EUR-3	EUR-4	China	Japan	America	Europe
CHI-N	0.68	0.18	0.09	—	—	—	—	—	—	0.84	0.16	—	—
CHI-S	0.16	0.73	0.05	0.02	0.02	—	—	—	—	0.86	0.05	0.09	—
JAP	0.07	0.07	0.72	0.13	—	—	—	—	—	0.09	0.80	0.09	0.02
NAM-1	—	—	0.06	0.82	0.03	—	—	—	0.03	—	0.06	0.88	0.03
NAM-2	—	0.03	0.03	0.23	0.63	—	0.03	0.03	—	0.03	—	0.91	0.06
EUR-1	—	—	—	0.24	0.11	0.45	—	0.16	0.05	—	—	0.42	0.58
EUR-2	0.02	—	0.02	0.34	0.10	0.06	0.24	0.10	0.10	0.02	0.02	0.52	0.44
EUR-3	—	—	0.03	0.25	0.05	0.08	0.05	0.50	0.05	—	0.03	0.38	0.60
EUR-4	0.02	—	—	0.27	0.10	0.02	0.06	0.15	0.38	0.02	—	0.42	0.56

Proportions of individuals in a population assigned with maximum probability to each population and region are given.
