# Supplementary material for: Population genetics of the Manila clam (Ruditapes philippinarum) introduced in North America and Europe
Source: Sci Rep. 2017 Jan 3;7:39745. doi: 10.1038/srep39745 (PMC5206634; doi:10.1038/srep39745)

Supplementary information

Population genetics of the Manila clam (*Ruditapes philippinarum*) introduced in North America and Europe

David Cordero, Marina Delgado, Baozhong Liu, Jennifer Ruesink, Carlos Saavedra

Contents:

- Supplementary Table S1

- Supplementary Table S2

- Supplementary Table S3

- Supplementary Table S4

-Supplementary Table S5

- Supplementary Fig. S1

Supplementary Table S1.- COI frequencies in the Manila clam populations considered in this study. The names of the populations sampled for this study are shown in all-capitals. Data for the remaining populations were obtained from references 33 and 36.

Haplotype	Regions																												Total
	China													Japan									America		Europe				
	Xia	Kia	Dal	Dl	Nb	Gz	Rs	Pt	Qd	CHI-N	Lz	Tj	CHI-S	Kag	Mik	Tok	Ari	Miy	Nan	Not	Akk	JAP	NAM-1	NAM-2	EUR-1	EUR-2	EUR-3	EUR-4	
A-8	-	-	-	-	-	-	-	-	-	-	-	-	-	-	-	-	-	-	-	1	-	-	-	-	-	-	-	-	1
A-9	-	-	-	-	-	-	-	-	4	-	-	6	-	1	1	1	1	-	-	2	4	2	9	9	3	5	2	2	52
A-10	-	-	-	-	-	-	-	-	-	-	-	-	-	-	-	-	-	-	-	2	-	-	-	-	-	-	-	-	2
A-11	-	-	-	-	-	-	1	-	1	-	-	1	-	2	1	2	1	-	-	-	-	3	1	4	9	6	16	8	56
A-12	-	-	-	-	-	-	-	-	-	-	-	-	-	-	-	-	1	-	-	-	-	-	-	-	-	-	-	-	1
A-13	-	-	-	-	-	-	-	-	-	-	-	-	-	1	-	-	1	-	-	-	-	-	-	-	-	-	-	-	2
A-14	-	-	-	-	-	-	-	-	-	-	-	-	-	-	-	-	1	-	-	-	-	-	-	-	-	-	-	-	1
A-15	-	-	-	-	-	-	-	-	-	-	-	-	-	-	-	-	-	1	-	-	-	-	-	-	-	-	-	-	1
A-16	-	-	-	-	-	-	-	-	-	-	-	-	-	-	-	-	-	1	-	-	-	-	-	-	-	-	-	-	1
A-17	-	-	-	-	-	-	-	-	-	-	-	-	-	-	-	-	-	1	-	-	-	-	-	-	-	-	-	-	1
A-18	-	-	-	-	-	-	-	-	-	-	-	-	-	-	-	-	-	-	1	-	-	-	-	-	-	-	-	-	1
A-19	-	-	-	-	-	-	-	-	-	-	-	-	-	-	-	-	-	-	1	-	-	-	-	-	-	-	-	-	1
A-20	-	-	-	-	-	-	-	-	-	-	-	-	-	-	-	-	-	-	1	-	-	-	-	-	-	-	-	-	1
A-21	-	-	-	-	-	-	-	-	-	-	-	-	-	-	-	-	-	-	1	-	-	-	-	-	-	-	-	-	1
A-22	-	-	-	-	-	-	-	-	1	-	-	-	-	-	-	-	-	-	1	-	-	-	-	-	-	-	-	-	2
A-23	-	-	-	-	-	-	-	-	-	-	-	-	-	-	2	-	-	-	-	-	-	-	7	12	1	6	6	8	42
A-24	-	-	-	-	-	-	-	-	-	-	-	-	-	-	1	1	-	-	-	-	-	-	-	-	-	-	-	-	2
A-25	-	-	-	-	-	-	-	-	-	-	-	-	-	-	-	1	-	-	-	-	-	1	-	-	-	-	-	-	2
A-35	-	-	-	-	-	-	-	-	2	-	-	2	-	-	-	-	-	-	-	-	-	-	-	-	-	-	-	-	4
A-37	-	-	-	-	-	-	1	-	2	-	-	2	-	-	-	-	-	-	-	-	-	3	-	-	-	-	-	-	8
A-38	-	-	-	-	-	-	-	-	2	-	-	-	-	-	-	-	-	-	-	-	-	-	-	-	-	-	-	-	2
A-41	-	-	-	-	-	-	-	-	1	-	-	-	-	-	-	-	-	-	-	-	-	-	-	-	-	-	-	-	1
A-42	-	-	-	-	-	-	-	-	-	-	-	-	-	-	-	-	-	-	-	-	1	-	-	-	-	-	-	-	1
A-43	-	-	-	-	-	-	-	-	-	-	-	-	-	-	-	-	-	-	-	-	2	-	-	-	-	-	-	-	2
A-44	-	-	-	-	-	-	-	-	-	-	-	-	-	-	-	-	-	-	-	-	1	-	-	-	-	-	-	-	1
A-45	-	-	-	-	-	-	-	-	-	-	-	-	-	-	-	-	-	-	-	-	1	-	-	-	-	-	-	-	1
A-46	-	-	-	-	-	-	-	-	-	-	-	-	-	-	-	-	-	-	-	-	1	-	-	-	-	-	-	-	1
A-47	-	-	-	-	-	-	-	-	-	-	-	-	-	-	-	-	-	-	-	-	1	-	-	-	-	-	-	-	1
A-48	-	-	-	-	-	-	-	-	-	-	-	-	-	-	-	-	-	-	-	-	1	-	-	-	-	-	-	-	1
A-60	-	-	-	-	-	-	-	-	-	-	-	1	-	-	-	-	-	-	-	-	-	-	-	-	-	-	-	-	1
A-61	-	-	-	-	-	-	-	-	-	-	-	1	-	-	-	-	-	-	-	-	-	-	-	-	-	-	-	-	1
A-62	-	-	-	-	-	-	-	-	-	-	-	1	-	-	-	-	-	-	-	-	-	-	-	-	-	-	-	-	1
A-63	-	-	-	-	-	-	-	-	-	-	-	1	-	-	-	-	-	-	-	-	-	2	-	-	-	-	-	-	3
A-68	-	-	-	-	-	-	-	-	-	-	-	-	-	-	-	-	-	-	-	-	-	1	-	-	-	-	-	-	1
A-73	-	-	-	-	-	-	-	-	-	-	-	-	-	-	-	-	-	-	-	-	-	1	-	-	-	-	-	-	1
A-74	-	-	-	-	-	-	-	-	-	-	-	-	-	-	-	-	-	-	-	-	-	1	-	-	-	-	-	-	1
A-75	-	-	-	-	-	-	-	-	-	-	-	-	-	-	-	-	-	-	-	-	-	1	-	-	-	-	-	-	1
A-76	-	-	-	-	-	-	-	-	-	-	-	-	-	-	-	-	-	-	-	-	-	-	-	-	1	2	3	1	7
A-77	-	-	-	-	-	-	-	-	-	-	-	-	-	-	-	-	-	-	-	-	-	-	-	-	13	2	7	8	30
A-78	-	-	-	-	-	-	-	-	-	-	-	-	-	-	-	-	-	-	-	-	-	-	-	-	-	1	-	-	1
A-94	-	-	-	-	-	-	-	-	-	-	-	-	-	-	-	-	-	-	-	-	-	-	1	-	-	-	-	-	1
A-95	-	-	-	-	-	-	-	-	-	-	-	-	-	-	-	-	-	-	-	-	-	-	1	-	-	-	-	-	1
A-96	-	-	-	-	-	-	-	-	-	-	-	-	-	-	-	-	-	-	-	-	-	-	1	-	-	-	-	-	1
A-97	-	-	-	-	-	-	-	-	-	-	-	-	-	-	-	-	-	-	-	-	-	-	2	-	-	-	-	-	2
A-98	-	-	-	-	-	-	-	-	-	-	-	-	-	-	-	-	-	-	-	-	-	-	1	-	-	-	-	-	1
A-99	-	-	-	-	-	-	-	-	-	-	-	-	-	-	-	-	-	-	-	-	-	-	1	-	-	-	-	-	1
A-100	-	-	-	-	-	-	-	-	-	-	-	-	-	-	-	-	-	-	-	-	-	-	1	-	-	-	-	-	1
A-101	-	-	-	-	-	-	-	-	-	-	-	-	-	-	-	-	-	-	-	-	-	-	1	-	-	-	-	-	1
A-102	-	-	-	-	-	-	-	-	-	-	-	-	-	-	-	-	-	-	-	-	-	-	1	-	-	-	-	-	1

Supplementary Table S1 (Continued).

Haplotype	Regions																													Total
	China													Japan									America		Europe					
	Xia	Kia	Dal	Dl	Nb	Gz	Rs	Pt	Qd	CHI-N	Lz	Tj	CHI-S	Kag	Mik	Tok	Ari	Miy	Nan	Not	Akk	JAP	NAM-1	NAM-2	EUR-1	EUR-2	EUR-3	EUR-4	
A-103	-	-	-	-	-	-	-	-	-	-	-	-	-	-	-	-	-	-	-	-	-	-	1	-	-	-	-	-	1
A-104	-	-	-	-	-	-	-	-	-	-	-	-	-	-	-	-	-	-	-	-	-	-	1	-	-	-	-	-	1
A-105	-	-	-	-	-	-	-	-	-	-	-	-	-	-	-	-	-	-	-	-	-	-	1	-	-	-	-	-	1
A-106	-	-	-	-	-	-	-	-	-	-	-	-	-	-	-	-	-	-	-	-	-	-	1	-	-	-	-	-	1
A-107	-	-	-	-	-	-	-	-	-	-	-	-	-	-	-	-	-	-	-	-	-	-	-	1	-	-	-	-	1
A-108	-	-	-	-	-	-	-	-	-	-	-	-	-	-	-	-	-	-	-	-	-	-	-	1	-	-	-	-	1
A-109	-	-	-	-	-	-	-	-	-	-	-	-	-	-	-	-	-	-	-	-	-	-	-	1	-	-	-	-	1
A-110	-	-	-	-	-	-	-	-	-	-	-	-	-	-	-	-	-	-	-	-	-	-	-	1	-	-	-	-	1
A-111	-	-	-	-	-	-	-	-	-	-	-	-	-	-	-	-	-	-	-	-	-	-	-	1	-	-	-	-	1
A-112	-	-	-	-	-	-	-	-	-	-	-	-	-	-	-	-	-	-	-	-	-	-	-	2	-	-	-	-	2
A-113	-	-	-	-	-	-	-	-	-	-	-	-	-	-	-	-	-	-	-	-	-	-	-	1	-	-	-	-	1
B-2	2	1	1	2	2	2	1	2	-	1	-	-	9	-	-	-	-	-	-	-	-	-	-	-	-	-	-	-	23
B-7	-	-	1	-	-	-	-	-	-	1	-	-	-	-	-	-	-	-	-	-	-	-	-	-	-	-	-	-	2
B-26	-	-	-	1	2	-	3	5	-	2	1	-	1	-	-	-	-	-	-	-	-	-	-	-	-	-	-	-	15
B-29	-	-	-	-	1	-	-	-	-	-	-	-	-	-	-	-	-	-	-	-	-	-	-	-	-	-	-	-	1
B-30	-	-	-	-	1	-	-	-	-	-	-	-	-	-	-	-	-	-	-	-	-	-	-	-	-	-	-	-	1
B-32	-	-	-	-	-	1	-	-	-	-	-	-	-	-	-	-	-	-	-	-	-	-	-	-	-	-	-	-	1
B-33	-	-	-	-	-	1	-	-	-	-	-	-	-	-	-	-	-	-	-	-	-	-	-	-	-	-	-	-	1
B-36	-	-	-	-	-	-	-	-	1	-	-	-	-	-	-	-	-	-	-	-	-	-	-	-	-	-	-	-	1
B-39	-	-	-	-	-	-	-	-	3	-	-	-	-	-	-	-	-	-	-	-	-	1	-	-	-	-	-	-	4
B-40	-	-	-	-	-	-	-	-	1	-	-	-	-	-	-	-	-	-	-	-	-	-	-	-	-	-	-	-	1
B-50	-	-	-	1	-	-	-	-	-	-	-	-	-	-	-	-	-	-	-	-	-	-	-	-	-	-	-	-	1
B-51	-	-	-	1	-	-	-	-	-	-	-	-	-	-	-	-	-	-	-	-	-	-	-	-	-	-	-	-	1
B-52	-	-	-	1	-	-	-	-	-	-	-	-	-	-	-	-	-	-	-	-	-	-	-	-	-	-	-	-	1
B-56	-	-	-	-	-	-	-	-	-	-	1	-	-	-	-	-	-	-	-	-	-	-	-	-	-	-	-	-	1
B-57	-	-	-	-	-	-	-	-	-	-	1	-	-	-	-	-	-	-	-	-	-	-	-	-	-	-	-	-	1
B-64	-	-	-	-	-	-	-	-	-	-	-	1	-	-	-	-	-	-	-	-	-	-	-	-	-	-	-	-	1
B-65	-	-	-	-	-	-	1	-	-	-	-	-	-	-	-	-	-	-	-	-	-	-	-	-	-	-	-	-	1
B-66	-	-	-	-	-	-	1	-	-	-	-	-	-	-	-	-	-	-	-	-	-	-	-	-	-	-	-	-	1
B-80	-	-	-	-	-	-	-	-	-	-	-	-	2	-	-	-	-	-	-	-	-	-	-	-	-	-	-	-	2
B-83	-	-	-	-	-	-	-	-	-	-	-	-	1	-	-	-	-	-	-	-	-	-	-	-	-	-	-	-	1
B-85	-	-	-	-	-	-	-	-	-	1	-	-	-	-	-	-	-	-	-	-	-	-	-	-	-	-	-	-	1
B-93	-	-	-	-	-	-	-	-	-	1	-	-	-	-	-	-	-	-	-	-	-	-	-	-	-	-	-	-	1
C-1	1	-	-	-	-	-	-	-	-	-	-	-	-	-	-	-	-	-	-	-	-	-	-	-	-	-	-	-	1
C-3	1	-	-	-	-	-	-	2	-	1	-	-	-	-	-	-	-	-	-	-	-	-	-	-	-	-	-	-	4
C-4	1	-	-	2	-	1	1	3	1	1	5	1	5	3	-	-	-	-	-	-	-	5	2	-	1	-	-	-	32
C-5	-	2	-	7	5	-	-	4	1	4	-	-	2	-	-	-	-	-	-	-	-	-	-	-	-	-	-	-	25
C-6	-	1	-	-	-	-	-	-	-	-	-	-	-	-	-	-	-	-	-	-	-	-	-	-	-	-	-	-	1
C-27	-	-	-	1	1	-	-	-	-	-	-	-	-	-	-	-	-	-	-	-	-	-	-	-	-	-	-	-	2
C-28	-	-	-	-	1	-	-	-	-	-	-	-	-	-	-	-	-	-	-	-	-	-	-	-	-	-	-	-	1
C-31	-	-	-	-	-	1	-	-	-	-	-	-	-	-	-	-	-	-	-	-	-	-	-	-	-	-	-	-	1
C-34	-	-	-	-	-	1	-	-	-	-	-	-	-	-	-	-	-	-	-	-	-	-	-	-	-	-	-	-	1
C-49	-	-	-	1	-	-	-	-	-	-	-	-	-	-	-	-	-	-	-	-	-	-	-	-	-	-	-	-	1
C-53	-	-	-	1	-	-	-	-	-	-	-	-	-	-	-	-	-	-	-	-	-	-	-	-	-	-	-	-	1
C-54	-	-	-	1	-	-	-	-	-	-	-	-	-	-	-	-	-	-	-	-	-	-	-	-	-	-	-	-	1
C-55	-	-	-	-	-	-	-	-	-	-	1	-	-	-	-	-	-	-	-	-	-	-	-	-	-	-	-	-	1
C-58	-	-	-	-	-	-	-	-	-	-	1	-	-	-	-	-	-	-	-	-	-	-	-	-	-	-	-	-	1
C-59	-	-	-	-	-	-	-	-	-	-	1	-	-	-	-	-	-	-	-	-	-	-	-	-	-	-	-	-	1
C-67	-	-	-	-	-	-	-	-	-	-	-	-	-	-	-	-	-	-	-	-	-	4	-	-	-	-	-	-	4
C-69	-	-	-	-	-	-	-	-	-	-	-	-	-	-	-	-	-	-	-	-	-	1	-	-	-	-	-	-	1
C-70	-	-	-	-	-	-	-	-	-	-	-	-	-	-	-	-	-	-	-	-	-	1	-	-	-	-	-	-	1
C-71	-	-	-	-	-	-	-	-	-	-	-	-	-	-	-	-	-	-	-	-	-	2	-	-	-	-	-	-	2
C-72	-	-	-	-	-	-	-	-	-	-	-	-	-	-	-	-	-	-	-	-	-	2	-	-	-	-	-	-	2

Supplementary Table S1 (Continued).

Haplotype	Regions																												Total
	China													Japan									America		Europe				
	Xia	Kia	Dal	Dl	Nb	Gz	Rs	Pt	Qd	CHI-N	Lz	Tj	CHI-S	Kag	Mik	Tok	Ari	Miy	Nan	Not	Akk	JAP	NAM-1	NAM-2	EUR-1	EUR-2	EUR-3	EUR-4	
C-79	-	-	-	-	-	-	-	-	-	-	-	-	1	-	-	-	-	-	-	-	-	-	-	-	-	-	-	-	1
C-81	-	-	-	-	-	-	-	-	-	-	-	-	1	-	-	-	-	-	-	-	-	-	-	-	-	-	-	-	1
C-82	-	-	-	-	-	-	-	-	-	-	-	-	1	-	-	-	-	-	-	-	-	-	-	-	-	-	-	-	1
C-84	-	-	-	-	-	-	-	-	-	1	-	-	-	-	-	-	-	-	-	-	-	-	-	-	-	-	-	-	1
C-86	-	-	-	-	-	-	-	-	-	2	-	-	-	-	-	-	-	-	-	-	-	-	-	-	-	-	-	-	2
C-87	-	-	-	-	-	-	-	-	-	1	-	-	-	-	-	-	-	-	-	-	-	-	-	-	-	-	-	-	1
C-88	-	-	-	-	-	-	-	-	-	1	-	-	-	-	-	-	-	-	-	-	-	-	-	-	-	-	-	-	1
C-89	-	-	-	-	-	-	-	-	-	2	-	-	-	-	-	-	-	-	-	-	-	-	-	-	-	-	-	-	2
C-90	-	-	-	-	-	-	-	-	-	1	-	-	-	-	-	-	-	-	-	-	-	-	-	-	-	-	-	-	1
C-91	-	-	-	-	-	-	-	-	-	1	-	-	-	-	-	-	-	-	-	-	-	-	-	-	-	-	-	-	1
C-92	-	-	-	-	-	-	-	-	-	1	-	-	-	-	-	-	-	-	-	-	-	-	-	-	-	-	-	-	1
Total	5	4	2	19	13	7	9	16	20	22	11	17	23	7	5	5	5	3	5	5	12	31	33	33	34	27	22	28	423

Supplementary Table S2 .- Mitochondrial COI genetic diversity and neutrality tests in 28 populations and four regions of *R. philippinarum* . *N*: number of sequences; *S*: segregating sites; *h*: number of haplotypes; H_d: haplotype diversity; π: nucleotide diversity estimated from pairwise differences; *D*: Tajima's neutrality tests. n.a.: not applicable. The names of 9 populations sampled especifically for this study appear as all-capitals. For the remaining populations, estimates were obtained from data provided in references 33 and 36.

	Region	Population	*N*	*S*	*h*	H_d		π (%)		Tajima's *D*
						All clades	*Clade A*	*All clades*	*Clade A*	
By populations										
	China									
		Dl1	2	1	2	1.00 ± 0.50	-	0.17 ± 0.09	-	n.a.
		Dl2	19	14	11	0.87 ± 0.07	-	0.66 ± 0.09	-	-0.392
		Tj	17	18	10	0.88 ± 0.07	0.84 ± 0.09	0.60 ± 0.15	0.35 ±0.07	-1.354
		Lz	11	12	7	0.82 ± 0.12	-	0.55 ± 0.12	-	-0.951
		Rs	9	15	7	0.92 ± 0.09	1.00 ± 0.50	0.83 ± 0.19	0.52 ± 0.26	-0.560
		Qd	20	21	12	0.94 ± 0.03	0.86 ± 0.06	1.03 ± 0.11	0.50 ± 0.09	0.076
		CHI-N	22	23	16	0.96 ± 0.03	-	0.72 ± 0.06	-	-1.277
		Kia	4	9	3	0.83 ± 0.22	-	0.83 ± 0.24	-	-0.154
		Nb	13	12	7	0.85 ± 0.09	-	0.79 ± 0.09	-	0.736
		Pt	16	8	5	0.83 ± 0.05	-	0.57 ± 0.05	-	1.330
		Xia	5	7	4	0.90 ± 0.16	-	0.62 ± 0.13	-	0.498
		Gz	7	11	6	0.95 ± 0.10	-	0.74 ± 0.17	-	-0.246
		CHI-S	23	13	9	0.81 ± 0.07	-	0.58 ± 0.06	-	-0.154
	Japan									
		Not	5	2	3	0.80 ± 0.16	0.80 ± 0.16	0.17 ± 0.05	0.17 ± 0.05	0.243
		Akk	12	12	8	0.89 ± 0.08	0.89 ± 0.08	0.39 ± 0.10	0.39 ± 0.10	-1.985*
		Tok	5	5	4	0.90 ± 0.16	0.90 ± 0.16	0.41 ± 0.09	0.41 ± 0.09	0.000
		Mik	5	5	4	0.90 ± 0.16	0.90 ± 0.16	0.45 ± 0.12	0.45 ± 0.12	0.562
		JAP	31	25	16	0.94 ± 0.02	0.92 ± 0.04	1.08 ± 0.06	0.49 ± 0.07	-0.133
		Kag	7	11	4	0.81 ± 0.13	0.83 ± 0.22	1.03 ± 0.18	0.26 ± 0.09	1.819
		Ari	5	8	5	1.00 ± 0.13	1.00 ± 0.13	0.61 ± 0.16	0.62 ± 0.16	-0.440
		Miy	3	7	3	1.00 ± 0.27	1.00 ± 0.27	0.80 ± 0.33	0.80 ± 0.33	n.a.
		Nan	5	7	5	1.00 ± 0.13	1.00 ± 0.13	0.55 ± 0.10	0.55 ± 0.10	-0.332
	N. America									
		NAM-1	33	27	17	0.89 ± 0.04	0.88 ± 0.04	0.65 ± 0.09	0.53 ± 0.06	-1.531
		NAM-2	33	13	10	0.79 ± 0.05	0.79 ± 0.05	0.45 ± 0.05	0.45 ± 0.05	-0.581
	Europe									
		EUR-1	28	13	6	0.69 ± 0.06	0.67 ± 0.06	0.29 ± 0.10	0.18 ± 0.05	-1.693
		EUR-2	22	8	6	0.82 ± 0.04	0.82 ± 0.04	0.44 ± 0.05	0.44 ± 0.05	0.577
		EUR-3	34	6	5	0.72 ± 0.06	0.72 ± 0.06	0.35 ± 0.05	0.35 ± 0.05	1.059
		EUR-4	27	6	5	0.76 ± 0.04	0.76 ± 0.04	0.40 ± 0.04	0.40 ± 0.04	1.414
By regions										
		China	168	60	59	0.93 ± 0.01	0.86 ± 0.04	0.95 ± 0.04	0.42 ± 0.06	-1.511
		Japan	78	42	38	0.95 ± 0.01	0.94 ± 0.02	0.91 ± 0.06	0.49 ± 0.04	-1.314
		N. America	66	34	24	0.84 ± 0.03	0.83 ± 0.03	0.55 ± 0.06	0.49 ± 0.04	-1.830*
		Europe	111	15	7	0.76 ± 0.02	0.75 ± 0.02	0.38 ± 0.03	0.35 ± 0.03	-0.636
By clades										
		Clade A	263	55	60	0.88 ± 0.01	-	0.45 ± 0.02	-	-2.124**
		Clade B	63	23	22	0.81 ± 0.04	-	0.32 ± 0.03	-	-1.935*
		Clade C	97	35	31	0.83 ± 0.03	-	0.37 ± 0.03	-	-2.163**

*, P<0.05;   **, P<0.01;   ***, P<0.001

Supplementary Table S3. Microsatellite allele frequencies at 7 loci for 9 *R. philippinarum* populations.

Locus	Allele (bp)	Population								
		CHI-N	CHI-S	JAP	NAM-1	NAM-2	EUR-1	EUR-2	EUR-3	EUR-4
A16	152	0.03	0.02	-	-	-	-	-	-	-
	156	-	-	0.03	-	-	-	0.01	-	-
	158	0.89	0.74	0.42	0.02	0.05	-	0.02	0.04	0.01
	160	0.03	0.13	0.04	0.18	0.15	0.16	0.20	0.15	0.16
	162	-	-	0.04	0.03	0.05	0.16	0.07	0.21	0.13
	164	-	-	0.14	0.02	-	-	0.01	-	-
	166	0.02	0.04	0.22	0.15	0.17	0.04	0.08	0.08	0.08
	168	0.02	0.06	0.06	0.20	0.20	0.21	0.23	0.14	0.19
	170	-	0.01	0.06	0.21	0.23	0.18	0.29	0.21	0.21
	172	-	-	-	0.11	0.09	0.08	0.03	0.12	0.04
	174	-	-	-	0.05	0.03	0.17	0.04	0.06	0.13
	176	-	-	-	0.02	0.02	-	-	-	0.02
	178	-	-	-	0.02	0.02	-	0.01	-	0.02
	180	-	-	-	0.02	0.02	-	-	-	0.01
	188	-	-	-	-	-	-	0.01	-	0.01
A24	157	0.02	-	0.10	-	-	-	-	-	-
	159	0.02	0.05	-	-	-	-	-	-	-
	163	0.88	0.77	0.55	0.72	0.62	0.75	0.80	0.76	0.82
	165	-	0.02	0.06	-	-	0.03	-	-	-
	167	0.05	0.14	0.13	-	0.03	0.03	0.04	0.01	-
	169	0.02	0.01	0.03	-	0.03	0.01	0.01	-	0.02
	171	-	-	-	-	0.06	0.08	0.06	0.08	0.05
	173	0.01	0.01	0.10	0.25	0.21	0.11	0.08	0.15	0.11
	175	-	-	0.03	0.02	0.05	-	0.01	-	-
	177	-	-	-	0.02	-	-	-	-	-
A54	239	-	-	-	-	0.04	-	-	-	-
	241	-	0.03	-	0.02	-	0.04	-	0.03	0.01
	243	0.01	0.03	-	-	-	0.03	0.02	-	0.04
	245	0.02	0.02	0.05	0.02	-	0.04	0.04	0.03	0.01
	247	0.30	0.35	0.41	0.41	0.40	0.21	0.23	0.40	0.38
	249	0.30	0.21	0.18	0.14	0.19	0.27	0.35	0.33	0.29
	251	0.03	0.03	0.04	0.16	0.06	0.09	0.07	0.07	0.02
	253	0.01	-	-	0.02	0.03	0.01	0.01	-	0.02
	255	-	0.02	0.04	0.02	-	-	0.01	-	-
	257	0.02	-	0.01	-	-	-	-	-	-
	259	-	-	0.01	-	-	-	-	-	-
	261	0.02	0.01	-	0.02	-	-	-	-	-
	267	-	-	-	-	-	-	-	-	0.01
	271	-	-	-	0.02	-	-	-	-	-
	null	0.28	0.28	0.27	0.20	0.29	0.30	0.27	0.16	0.22
A62	208	-	-	0.04	-	-	-	-	-	-
	216	0.05	0.04	0.01	-	-	-	-	-	-
	218	0.05	0.03	0.03	0.02	-	-	-	-	-
	220	0.09	0.05	0.04	0.02	-	0.02	0.02	0.01	0.02
	222	0.07	0.03	0.01	-	0.03	-	0.01	-	-
	224	0.01	-	0.08	0.09	0.05	0.09	0.05	0.06	0.07
	226	0.09	0.18	-	0.02	0.03	-	0.01	-	0.02
	228	0.08	0.09	0.21	0.33	0.32	0.34	0.32	0.31	0.26
	230	0.16	0.46	0.26	0.30	0.42	0.27	0.29	0.30	0.34
	232	0.11	0.04	0.04	0.05	0.03	0.15	0.13	0.10	0.09
	234	0.12	-	0.19	0.12	0.05	0.12	0.11	0.19	0.17
	236	0.04	0.05	0.06	0.03	-	0.02	0.02	-	0.01
	238	0.05	0.01	-	-	-	-	0.01	-	-
	240	0.04	0.03	0.01	-	0.02	-	-	-	-
	242	0.01	0.01	0.01	-	0.02	-	-	0.01	-
	244	0.02	-	-	0.02	-	-	0.01	-	0.01
	246	0.01	-	-	-	-	-	-	-	-
	248	-	-	0.01	-	0.02	-	-	-	-
	250	-	-	-	0.02	0.02	0.02	0.01	0.01	-
	254	0.01	-	-	-	-	-	-	-	-

Supplementary Table S3 (Continued).

Locus	Allele (bp)	Population								
		CHI-N	CHI-S	JAP	NAM-1	NAM-2	EUR-1	EUR-2	EUR-3	EUR-4
A64	106	0.01	-	0.02	-	-	-	-	-	-
	110	0.11	0.05	0.09	0.08	0.08	0.04	0.03	-	0.03
	114	0.01	-	-	-	-	-	0.03	-	-
	116	0.01	0.01	-	-	-	0.01	-	0.01	0.01
	118	0.25	0.31	0.23	0.24	0.30	0.28	0.25	0.28	0.27
	120	0.11	0.10	0.09	0.05	0.11	0.01	0.05	0.01	0.02
	122	0.03	-	-	-	-	-	-	-	-
	124	0.01	0.01	0.01	0.05	0.03	0.07	0.07	0.08	0.03
	126	0.35	0.44	0.43	0.50	0.42	0.49	0.47	0.43	0.46
	128	-	-	-	0.03	-	0.01	0.01	0.01	0.01
	130	0.02	-	-	-	-	-	-	-	0.01
	132	0.01	0.01	-	0.02	0.02	-	0.01	0.03	0.01
	134	0.01	0.01	0.02	0.03	0.03	-	-	-	-
	136	-	0.01	0.05	-	0.02	0.01	-	0.01	-
	138	0.02	0.01	-	-	-	0.01	0.01	0.08	0.05
	140	-	-	-	-	-	0.03	0.01	0.01	-
	142	0.01	0.02	-	-	-	0.03	0.06	0.06	0.09
	144	-	0.01	0.01	-	-	-	-	-	-
	146	-	-	0.04	-	-	-	-	-	-
	148	-	-	0.01	0.02	-	-	-	-	-
	150	0.01	-	-	-	-	-	-	-	-
K8	144	-	-	-	0.03	-	-	-	-	-
	154	-	-	0.01	-	-	-	0.01	-	-
	160	0.04	0.06	-	-	-	-	-	-	0.03
	162	0.38	0.24	0.26	0.15	0.24	0.14	0.20	0.19	0.15
	164	0.35	0.31	0.50	0.46	0.49	0.49	0.53	0.38	0.58
	166	0.15	0.22	0.17	0.18	0.17	0.26	0.18	0.31	0.16
	168	0.06	0.08	0.05	0.09	0.09	0.04	0.08	0.09	0.06
	170	0.01	0.01	0.01	0.02	0.02	0.03	-	0.01	0.01
	174	0.01	0.01	-	-	-	0.04	-	0.03	0.01
	null	-	0.06	-	0.08	-	-	-	-	-
K22	175	-	-	-	-	0.02	0.06	-	-	-
	178	-	-	-	-	0.05	0.01	0.01	-	-
	181	0.03	-	0.02	0.08	0.09	0.17	0.07	0.16	0.11
	184	-	0.01	0.01	-	0.05	0.03	0.04	0.05	0.05
	199	-	-	-	-	-	-	-	-	0.01
	205	-	-	-	-	0.02	-	-	-	-
	214	-	0.01	-	-	0.03	-	-	-	-
	217	-	-	-	0.02	-	-	-	0.03	-
	220	0.01	-	-	-	0.02	-	-	-	-
	223	0.03	-	-	-	-	-	0.02	-	0.02
	226	0.06	0.06	0.04	0.09	0.03	0.11	0.12	0.15	0.11
	229	-	0.05	0.02	0.05	0.03	-	-	-	0.03
	232	0.04	0.05	0.02	0.05	-	-	0.01	0.03	0.08
	235	0.06	0.06	0.08	0.08	0.08	-	0.04	0.05	0.01
	238	0.12	0.12	0.08	0.03	0.11	0.03	0.05	0.04	0.08
	241	0.08	0.14	0.07	0.09	-	0.04	0.06	0.03	0.03
	244	0.08	0.08	0.10	0.09	-	0.07	0.07	0.10	0.10
	247	0.10	0.07	0.07	0.03	0.03	0.01	0.05	0.01	0.02
	250	0.14	0.08	0.03	0.06	0.03	-	0.01	0.03	0.02
	253	0.03	0.04	0.02	0.03	0.03	-	0.01	0.01	-
	256	0.03	0.04	0.02	0.03	0.02	0.04	0.02	0.03	0.02
	259	0.04	0.04	-	0.03	0.02	0.04	0.02	-	-
	262	-	-	-	0.03	-	-	-	-	-
	265	-	-	-	0.03	-	-	-	-	-
	289	-	0.01	-	-	0.02	0.03	-	-	-
	null	0.17	0.16	0.42	0.20	0.36	0.34	0.39	0.28	0.30

Supplementary Table S4. Genetic diversity and F_{IS} of microsatellite markers in 9 populations of *R. philippinarum*. 2N: number of gene copies; F_{IS}: inbreeding coefficient; H_e: heterozygosity; N_a: number of alleles; A: allelic richness.

		CHI-N	CHI-S	JAP	NAM-1	NAM-2	EUR-1	EUR-2	EUR-3	EUR-4
A16	2N	88	86	72	66	66	76	100	78	96
	F_{IS}	-0.066	0.026	-0.064	0.119	-0.097	0.131	-0.004	-0.046	-0.012*
	H_e	0.213	0.430	0.758	0.858	0.858	0.846	0.817	0.858	0.865
	N_a	5	6	8	12	11	7	12	8	12
	A	4.78	5.62	7.98	11.69	10.82	7.00	10.27	7.99	10.68
A24	2N	88	88	78	64	66	76	84	74	94
	F_{IS}	-0.073	-0.061	-0.047*	-0.324	-0.281	-0.055	0.064	-0.208	-0.140
	H_e	0.233	0.386	0.662	0.427	0.570	0.424	0.356	0.404	0.318
	N_a	6	6	7	4	6	6	6	4	4
	A	5.44	5.32	6.92	3.94	5.99	5.75	5.46	3.84	3.88
A54	2N	88	88	84	64	70	70	100	76	96
	F_{IS}	0.123***	-0.021***	-0.007*	0.097	-0.100	-0.013*	-0.011*	0.118	0.118***
	H_e	0.750	0.757	0.733	0.760	0.728	0.790	0.752	0.715	0.731
	N_a	9	9	8	10	6	8	8	6	9
	A	8.13	8.46	7.44	9.81	5.99	7.87	7.08	5.94	7.68
A62	2N	86	80	80	66	62	68	92	80	96
	F_{IS}	0.015	-0.072	0.115	0.149	0.019	0.024	0.037	-0.036	-0.043
	H_e	0.921	0.747	0.846	0.782	0.723	0.783	0.790	0.772	0.779
	N_a	17	12	14	11	11	8	12	8	9
	A	15.75	11.38	12.80	10.69	11.00	7.74	10.16	7.33	8.05
A64	2N	88	88	82	66	66	74	98	80	92
	F_{IS}	0.026	-0.100	-0.004	0.078	0.075	-0.069	-0.002	0.084	-0.132*
	He	0.793	0.704	0.753	0.690	0.720	0.683	0.713	0.736	0.712
	N_a	15	12	11	9	8	11	11	11	11
	A	12.44	9.84	10.14	8.87	7.87	10.14	9.43	9.83	9.52
K8	2N	80	78	78	66	66	72	76	80	80
	F_{IS}	0.131	0.065*	0.110	0.092	0.065	0.103	0.155	0.038	0.160
	H_e	0.718	0.794	0.662	0.733	0.680	0.680	0.652	0.727	0.623
	N_a	7	8	6	7	5	6	5	6	7
	A	6.54	7.59	5.59	6.94	4.94	5.98	4.82	5.73	6.50
K22	2N	78	86	92	66	66	70	98	74	92
	F_{IS}	0.076	0.140*	-0.013	0.183***	0.104***	-0.027*	0.003	0.124***	-0.006
	H_e	0.914	0.918	0.794	0.924	0.844	0.835	0.819	0.862	0.865
	N_a	15	16	14	17	18	13	16	14	15
	A	14.62	15.09	13.11	16.92	17.62	12.73	14.09	13.55	13.86
Total	χ^2	Infinity	39.2268	24.5613	Infinity	Infinity	26.8067	26.2667	28.6358	Infinity
	χ^2 Prob	High. sign.	0.0003	0.0392	High. sign.	High. sign.	0.0204	0.0239	0.0117	High. sign.
	Mean H_e	0.649	0.677	0.744	0.739	0.732	0.720	0.700	0.725	0.699
	S.D. (H_e)	0.301	0.196	0.067	0.158	0.098	0.146	0.163	0.154	0.189
	Mean N_a	10.6	9.9	9.7	10.0	9.3	8.4	10.0	8.1	9.6
	S.D. (N_a)	5.0	3.7	3.3	4.1	4.5	2.6	3.9	3.4	3.6
	Mean A	9.67	9.04	9.14	9.84	9.18	8.17	8.76	7.74	8.60
	S.D. (A)	4.53	3.43	2.94	4.05	4.42	2.48	3.22	3.18	3.18

* $P < 0.05$; ** $P < 0.01$; *** $P < 0.001$.

Supplementary Table S5. Join distribution of mitochondrial clades and microsatellite cluster 3 obtained from Structure's Bayesian inference, in the population of Japan.

Individual	COI clade	Cluster 3 estimated membership coefficient
JAP1	C	0.111
JAP2	C	0.945
JAP4	A	0.390
JAP5	A	0.513
JAP6	A	0.569
JAP7	A	0.822
JAP8	A	0.315
JAP13	C	0.091
JAP15	C	0.280
JAP16	A	0.415
JAP21	C	0.843
JAP25	B	0.145
JAP26	C	0.948
JAP27	C	0.949
JAP28	A	0.304
JAP30	C	0.858
JAP32	C	0.726
JAP35	C	0.060
JAP36	C	0.439
JAP38	C	0.016
JAP39	A	0.418
JAP40	A	0.705
JAP41	C	0.255
JAP42	A	0.1195
JAP43	A	0.0395
JAP44	A	0.156
JAP45	A	0.376
JAP46	C	0.102

Figure S1.- Bayesian model-based cluster analysis of individual genotypes at seven microsatellite markers in 9 populations of Manila clam. a: Diagram of posterior probability of the microsatellite data according to Evanno et al. for K = 1 to K = 10. b: Diagram of posterior probability of the microsatellite data according to Pritchard et al. for K = 1 to K = 10.

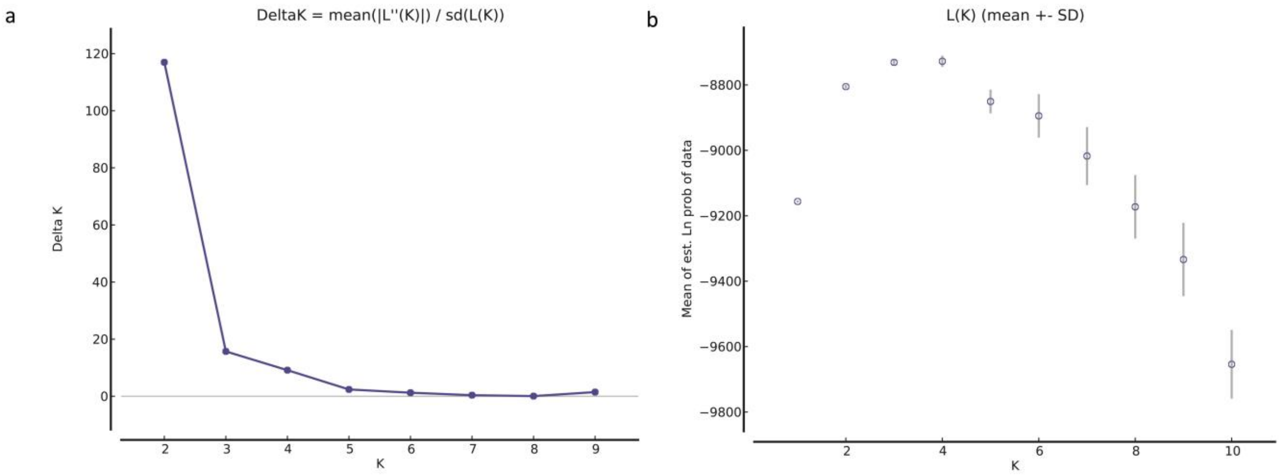

Supplement: Supplementary Tables and Figures [file srep39745-s1.pdf]
